# Particle Mobility Analysis Using Deep Learning and the Moment Scaling Spectrum

**DOI:** 10.1038/s41598-019-53663-8

**Published:** 2019-11-20

**Authors:** Marloes Arts, Ihor Smal, Maarten W. Paul, Claire Wyman, Erik Meijering

**Affiliations:** 1 Department of Medical Informatics, Erasmus University Medical Center, Rotterdam, The Netherlands; 2 Faculty of Applied Sciences, Delft University of Technology, Delft, The Netherlands; 3 Department of Radiology, Erasmus University Medical Center, Rotterdam, The Netherlands; 4 Department of Geoscience and Remote Sensing, Delft University of Technology, Delft, The Netherlands; 5 Department of Molecular Genetics, Oncode Institute, Erasmus University Medical Center, Rotterdam, The Netherlands; 6 Department of Radiation Oncology, Erasmus University Medical Center, Rotterdam, The Netherlands; 7 School of Computer Science and Engineering, University of New South Wales, Sydney, Australia; 8 Graduate School of Biomedical Engineering, University of New South Wales, Sydney, Australia

**Keywords:** Image processing, Machine learning, Protein analysis, Protein function predictions, Sequence annotation, Software, Statistical methods, Protein transport, Cell biology, Computational biology and bioinformatics, Molecular biology, Single-molecule biophysics, Software, Biological physics

## Abstract

Quantitative analysis of dynamic processes in living cells using time-lapse microscopy requires not only accurate tracking of every particle in the images, but also reliable extraction of biologically relevant parameters from the resulting trajectories. Whereas many methods exist to perform the tracking task, there is still a lack of robust solutions for subsequent parameter extraction and analysis. Here a novel method is presented to address this need. It uses for the first time a deep learning approach to segment single particle trajectories into consistent tracklets (trajectory segments that exhibit one type of motion) and then performs moment scaling spectrum analysis of the tracklets to estimate the number of mobility classes and their associated parameters, providing rich fundamental knowledge about the behavior of the particles under study. Experiments on in-house datasets as well as publicly available particle tracking data for a wide range of proteins with different dynamic behavior demonstrate the broad applicability of the method.

## Introduction

Single particle tracking in live cell fluorescence microscopy imaging data serves as a powerful tool to study the dynamics of a wide range of different particles. Here, “particle” is a generic term that can, amongst others, refer to small fluorophores, single molecules, macromolecular complexes, viruses, organelles or microspheres^1,2^. Consequently, single particle tracking (SPT) can be broadly applied in microrheology^3–6^ as well as in studying dynamic processes in live cells. Examples of such processes are microtubule assembly and disassembly^7^, cell migration governed by focal adhesions^8^, membrane dynamics^9^, intracellular transport^10^, chromatin assembly and gene transcription^11^, genome maintenance^12,13^, and virus trafficking^14^. Since manual tracking is subjective and becomes quite cumbersome for large datasets, automated tracking is preferred^15,16^. Many different software tools are available for SPT and new methods are still being developed^17^. SPT results in a series of coordinates over time for every single particle (also called “trajectories”), but by itself does not provide direct insights into the dynamic process of interest.

In order to relate trajectories of individual particles to the behavior of the population, mobility patterns must be analyzed in an automated, unbiased and statistically relevant way. As molecular behavior is commonly linked to function and structure, mobility analysis is connected to a deeper understanding of the associated biological process. The goal is to quantify behavior by determining physical properties of the particle of interest, such as velocity, processivity, confinement or spatial distribution^18,19^. Additional biological insights into the dynamic behavior of populations with mixed mobility can be provided by determining the relative fractions of particles in different functional states under varying conditions.

There are several approaches to this type of analysis, each with its own drawbacks. Methods based on single time steps, such as hidden Markov modeling (HMM)^20–23^ and probability density function (PDF) or cumulative distribution function (CDF) fitting, are problematic for detecting motion types that exhibit patterns over longer time-scales (Supplementary Note 1). There are also methods that use rolling windows of multiple time-points for classification. The main methods in this category are based on machine learning^24,25^ and, most commonly used in biological research, mean square displacement (MSD) analysis^9,26–28^ (Supplementary Note 2). A drawback of these methods is that a set window size introduces a trade-off between sensitivity and accuracy. Moreover, MSD-based methods are mostly limited to quantitative analysis of particles that exhibit pure diffusion, while in practice confined (subdiffusive) motion and highly correlated (superdiffusive) motion are quite common.

A critical limitation is that most of these methods are not able to detect switching from one behavior type to another within single trajectories, while change in behavior is the core of biological function. One approach to capturing this transient behavior uses image segmentation to distinguish between free motion and trapping of molecules^29^. In this method, the trapping state is characterized by the accumulation of trajectory segments, leading to a denser cloud in the image. However, in applications such as ours, this type of approach leads to inaccuracies when the times spent in each state become relatively short, or when trajectories are not long enough or do not form compact and well defined regions where particles are trapped.

More recently, a method was developed that uses divide-and-conquer classification (where trajectories first get an initial segmentation that is refined in subsequent steps) in combination with the moment scaling spectrum (MSS)^30^, an advanced measure for random motion characterization that has also been used in a variety of other motion studies^31–33^. By uncoupling segmentation and further motion analysis, this method allows to detect different types of motion as well as mobility switches. However, the number and the location of switching points are not always determined accurately, segmentation takes multiple steps, and there is a higher probability of misclassification for shorter trajectories^30^.

In this paper, a novel general method is presented to robustly analyze particle trajectories, providing information about the type of motion, associated parameters, and switching behavior. Here, particle trajectories are analyzed using state-of-the-art deep learning techniques in combination with advanced post-processing. The proposed method consists of two components and will henceforth be referred to as DL-MSS (Deep Learning followed by Moment Scaling Spectrum analysis).

Firstly, a deep learning (DL) neural network is trained with simulated data containing trajectories that switch between different types of mobility. This self-contained deep learning approach does not require any specific modelling or manual parameter tuning. Even though one could also use manually annotated real data, if available, the approach of using simulated trajectories to estimate the dynamics of real systems has been shown to be fruitful previously^34^. The trained network is applied to real microscopy imaging data to segment trajectories into segments, referred to as “tracklets”, that exhibit the same type of motion.

Subsequently, these tracklets are further analyzed using the moment scaling spectrum (MSS) and clustered according to their diffusion constant and type of motion to determine parameters associated with each class of mobility. The concept of the MSS is not new in more theoretical fields, but its practical application is overshadowed by the simpler MSD analysis, which can provide only a limited understanding of the underlying random behavior. MSS is a very robust tool to analyze and understand what modes of motion are present in a dataset, and implicitly contains other frequently used methods such as MSD and correlation between subsequent displacements^35,36^.

DL-MSS is able to perform segmentation in a single step without being limited to any trajectory length and returns a number of mobility classes with their associated parameters, providing fundamental knowledge about the behavior of the particle in question. Since this method can separate different populations in a dataset, DL-MSS makes it possible to compare the collective mobility of a specific type of molecule at different conditions.

There are many potential applications of single particle tracking (SPT) and trajectory analysis using DL-MSS. In this article, the focus is on mobility patterns of different nuclear proteins that exhibit multiple types of random walk-type behavior. DL-MSS was inspired by observable switches in motion for breast cancer susceptibility protein 2 (BRCA2). This large, multifunctional protein is most well-known for its role in the repair of double strand breaks (DSBs) in DNA^37,38^. Since DSBs can be introduced in live cells artificially through ionizing radiation^39^, BRCA2 mobility pattern analysis provides a nice showcase to detect behavioral changes upon DNA damage induction. As control datasets, histone protein H2B and nuclear localization signal (NLS) were used, as these molecules are characterized as mostly stuck or mostly free, respectively. The immobile H2B dataset can simultaneously be used to confirm that global movements of the cell are negligible compared to the local movement of single molecules^12,40,41^. Moreover, four publicly available datasets (generously provided by the authors of^42^) were used that contain trajectories of several proteins that are expected to exhibit different types of mobility, ranging from immobile to freely diffusing. DL-MSS was used successfully to classify and analyze all these different datasets in accordance with expected results.

## Results

### Deep learning neural network

**Figure 1:**
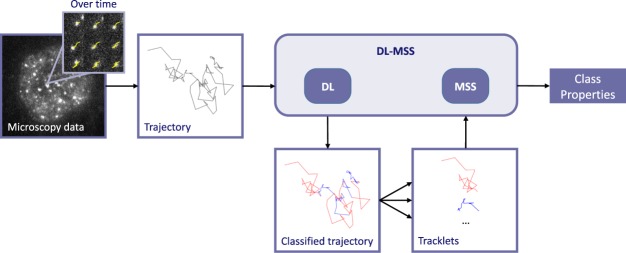
Overview of the DL-MSS method. Automatic tracking software is used to obtain single molecule trajectories from fluorescence microscopy data. A trained deep learning (DL) neural network is applied to these trajectories to segment them into “tracklets” of consecutive track points that were classified to have the same type of mobility. Tracklets are further analyzed using the moment scaling spectrum (MSS) to acquire the properties associated with each class.

DL-MSS consists of two elements: a deep learning part followed by a post-processing part (Fig. 1**)**. A long short term memory (LSTM) deep learning recurrent neural network was used for trajectory segmentation. This type of model was chosen because LSTM networks are known to be flexible to input size (which in this study depends on the trajectory length) and to be able to retain information over longer timescales^43–45^ (Supplementary Note 3). This network was trained with simulated trajectories that switch between three mobility classes: one fast diffusing state (diffusion constant 1.0 μm^2^/s), one slow diffusing state (diffusion constant 1.0 μm^2^/s) and one immobile state. The immobile state is chosen to reflect the situation where a molecule is “stuck”, e.g. where it is impossible to distinguish between motion of the molecule, the movement of the cell^41^ and the localization error of the molecules. These classes were chosen to reflect the mobility patterns of fluorescently labeled BRCA2, which served as the incentive to develop DL-MSS and showed at least two mobility classes, namely immobile and diffusing^12^. One extra class was added to increase the flexibility of the model without introducing overfitting to extra clusters that do not provide useful information. As the proposed deep learning network will not inadvertently detect motion types that are not actually present (Supplementary Note 4) and potential additional mobility classes can be detected later on in MSS analysis (Supplementary Note 5), this three-state model provides a simple yet flexible basis for classification. On simulated three-state mobility data, the trained network achieved an accuracy of 0.94 on the training set and an accuracy of 0.92 on the testing set (Online Methods), out of a maximum accuracy of 1. The trained network can be applied to unseen simulated data as well as trajectories extracted from real microscopy data and classifies these trajectories per time step for any length of trajectory (Supplementary Note 6). The same trained network was used for classification of all datasets mentioned in this paper.

### Moment scaling spectrum analysis

Segmented trajectories produce so-called “tracklets”, which are segments that are classified to one of the three states. These tracklets are further analyzed using the moment scaling spectrum (MSS). As opposed to classical methods such as MSD-based analysis, which makes use of only the second moment (*x*^2^ ∼ *τ* with *x* position and *τ* time step), MSS utilizes higher order moments^27,35^:

1 $$\langle {|x|}^{p}\rangle (\tau )=\frac{1}{N}\mathop{\sum }\limits_{n=1}^{N}\mathop{\sum }\limits_{t=1}^{{T}_{n}-\tau }{|{x}_{n}(t+\tau )-{x}_{n}(t)|}^{p}$$

where *N* is the number of trajectories, *T*_*n*_ is the duration of trajectory *n*, *τ* is the time step, *x*_*n*_(*t*) is the position of the *n*^*th*^ particle at time *t*, and *p* is the moment order. This means that *x*^*p*^ ∼ *τ*^*γp*^, where the plot of *γ*_*p*_ versus *p* gives the MSS. The slope of the MSS, denoted *S*_*MSS*_, indicates the motion type of the tracklet. In this spectrum, *S*_*MSS*_ = 0.5 represents pure diffusion, $0 < {S}_{MSS} < 0.5$ represents restricted motion, and $0.5 < {S}_{MSS} < 1.0$ represents more directed motion^30^ (Supplementary Note 7). The *S*_*MSS*_ can be calculated along with the diffusion constant *D* (to distinguish between “faster” and “slower” motion, Supplementary Note 8) for every tracklet in order to obtain a scatterplot of all tracklets together in *S*_*MSS*_-*D* space^46^. This procedure yields clusters of tracklets with the same kind of mobility, showing the properties of the different classes of tracklets. Because MSS analysis is less reliable for shorter tracklets, only tracklets of more than ten time frames are used for clustering (Supplementary Note 9). However, as MSS analysis is used only to determine the properties for clusters of tracklets with the same classification label, these properties can still be assigned to shorter tracklets as well, because they were classified by the deep learning neural network to have the same type of mobility as the longer tracks. Note that this method does not deny there can be more than three classes of mobility in a given dataset. Clusters can be subdivided into multiple classes recursively. DL-MSS aims to find the major clusters of motion so the proportions of tracklets in those clusters can be compared between different datasets.

### Showcase 1: BRCA2 behavioral change upon treatment with ionizing radiation

The first showcase for the application of DL-MSS is the response of BRCA2 protein mobility upon treatment of the cells with ionizing radiation (IR), which introduces DSBs into DNA. BRCA2 plays an important role in the repair of DSBs, and is known to accumulate at nuclear sites of DNA damage^39,47^. Consequently, the corresponding hypothesis is that more BRCA2 molecules should become immobile upon IR treatment compared to untreated cells^12^.

**Figure 2:**
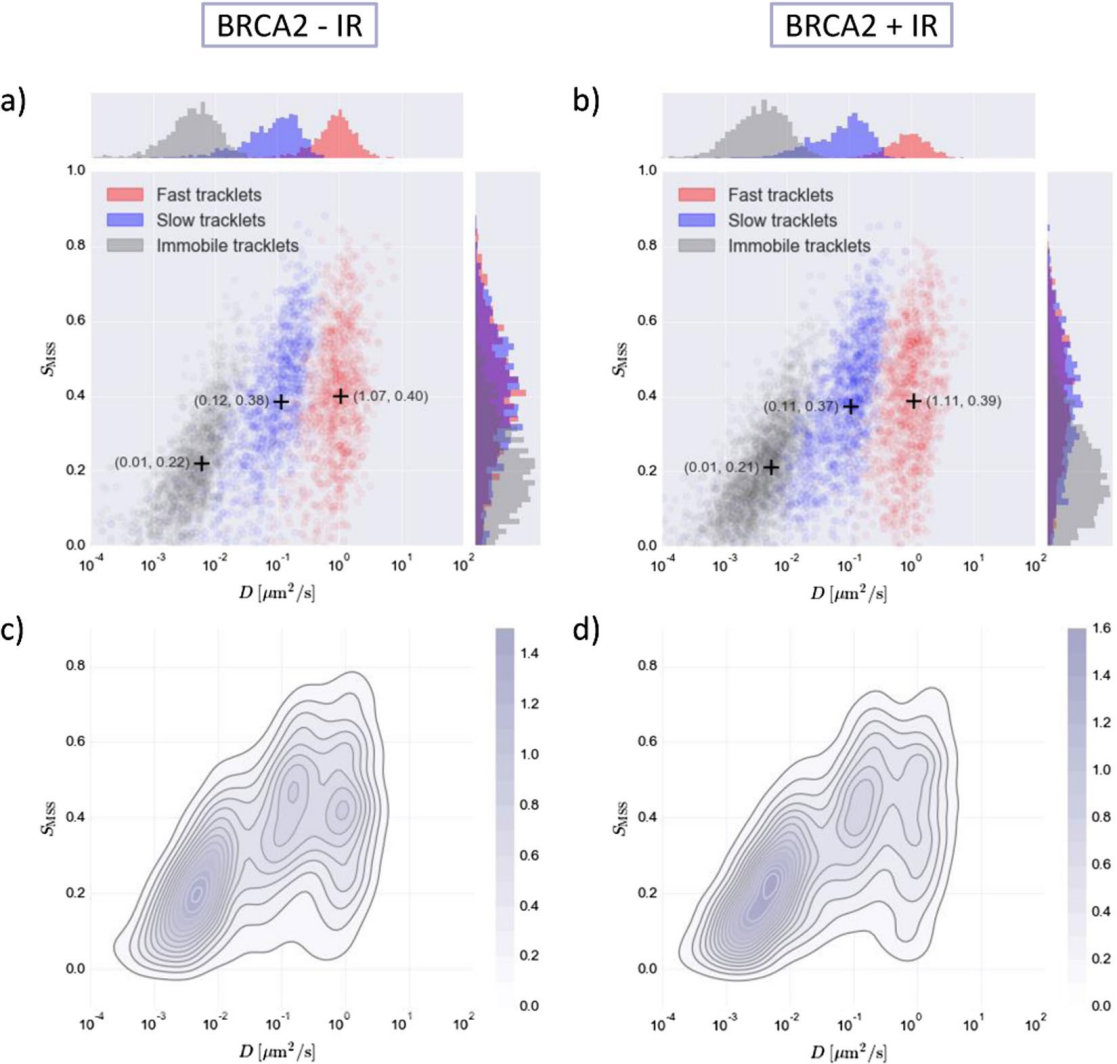
*S*_*MSS*_ versus *D* plots for the BRCA2 protein without and with ionizing radiation (IR). (**a,b**) scatterplot for BRCA2 –IR/BRCA2 + IR where red, blue and grey color coding corresponds to fast, slow and immobile tracklets, respectively. Histograms on the sides show the distributions of the tracklets in different clusters relative to each other for the different axes. Cluster means are indicated by the + symbol. (**c,d**) kernel density estimation plot for BRCA2 –IR/BRCA2 + IR, color intensity indicates density (see colorbar).

From the *S*_*MSS*_ versus *D* scatterplot of wildtype BRCA2 without any treatment (Fig. 2a), it is clear that the three-state mobility model fits the data well; the clusters of data points with common mobility characteristics are well defined, well sorted by class (each cluster contains only one color), and well separated. Moreover, no extra clusters are visible, indicating there are no additional mobility classes. The location of each cluster mean (indicated with “+” in Fig. 2a) in *S*_*MSS*_-*D* space specifies the properties of the corresponding class of tracklets. For BRCA2 without IR, DL-MSS yields three motion types: the first is very slow and immobile, the second is slow and close to free diffusion, and the third is fast and close to free diffusion as well. The immobile cluster presumably reflects protein localized to perform its repair function. The possibility to detect multiple mobile states (in this case slow and fast diffusion) is important because these different states can be biologically relevant, as proteins can be modified and can also interact with other molecules and structures in the cell.

The same type of scatterplot for BRCA2 tracklets from cells treated with IR (Fig. 2b) shows that even though there is very little variation in the location of the data clusters in *S*_*MSS*_-*D* space, their relative fractions change upon IR-treatment. This means that particle mobility characteristics do not change but the portion of particles in the different classes does change, which becomes even more clear when comparing the kernel density estimation (KDE) maps (Fig. 2c,d). These density maps show a shift from the diffusive states (mainly the fast diffusive state) to the immobile state after inducing DSBs, corresponding to the idea that more damage sites require more BRCA2 molecules to become “stuck” in order to perform their task. DL-MSS serves as a tool to successfully detect this behavioral change in a unique way, as it provides information about how fast the molecules move around as well as the specific type of motion. What is striking about this type of analysis is that it reveals how the relative intensities of the data clusters change rather than the cluster locations.

### Showcase 2: Unimodal mobility of histone protein H2B-HaloTag and nuclear localization signal (HaloTag-NLS)

**Figure 3:**
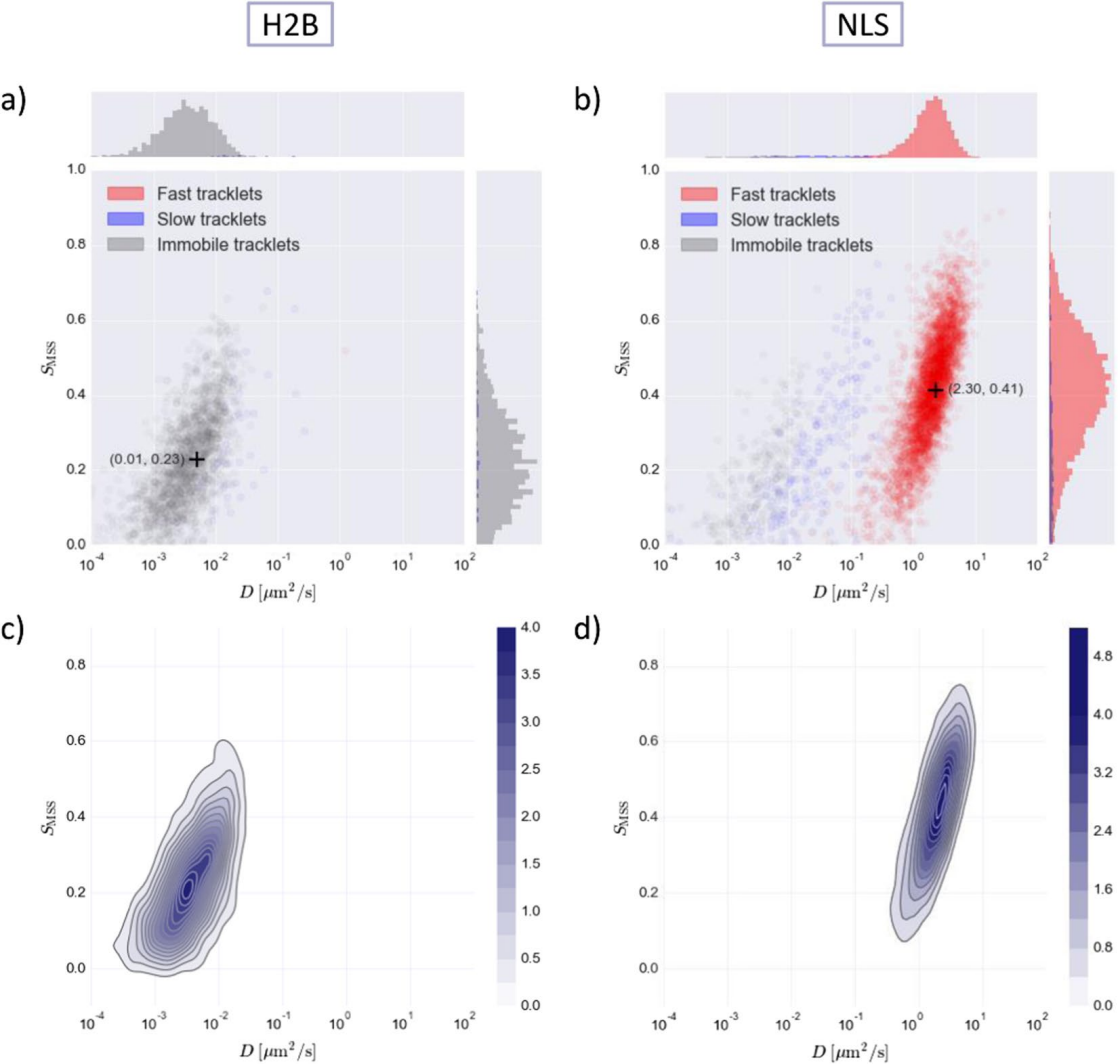
*S*_*MSS*_ versus *D* plots for the H2B protein and NLS. HaloTag was used for tracking. (**a,b**) scatterplot for H2B and NLS where red, blue and grey color coding corresponds to fast, slow and immobile tracklets, respectively. Histograms on the sides show the distribution of tracklets in the clusters relative to each other for the different axes. Cluster means are indicated by the + symbol. (**c,d**) kernel density estimation plot for H2B and NLS, color intensity indicates density (see colorbar).

Of course, not every molecule exhibits three types of mobility. In order to test whether or not DL-MSS is prone to overfitting, trajectories were analyzed for two molecules for which the behavior is known to be very simple. HaloTag labelled histone protein H2B and a nuclear localization signal (HaloTag-NLS) were chosen for this purpose because they are known to be predominantly immobile and fast diffusing, respectively^48,49^. DL-MSS finds only an immobile cluster for H2B (Fig. 3a,c) and almost exclusively finds fast diffusing tracklets for NLS (Fig. 3b,d), where the corresponding diffusion constant is considerably higher than that of the fast diffusive population of BRCA2 (Fig. 2). This result was obtained using the same network as for the first showcase, trained on the same simulated three-state mobility data. These results not only show that DL-MSS identifies the expected clusters for these control datasets, but also that this method does not find mobility classes that are not present in the data and that clusters are not necessarily bound to specific locations.

### Showcase 3: Publicly available datasets for H2B, CTCF, Sox2 and 3 × NLS

**Figure 4:**
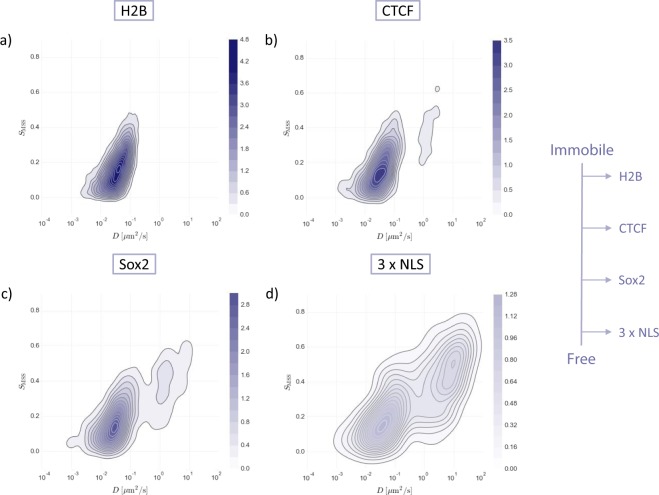
Kernel density estimation plots for Spot-On datasets that range from mainly immobile to mainly free. Color intensity indicates density (see colorbar).

Finally, DL-MSS was applied to four datasets that were made publicly available by the authors of^42^, which were imaged and tracked in a different way than the datasets analyzed above (Online Methods). This was done in order to demonstrate that the applicability of DL-MSS is not limited to our own type of imaging data, particle dynamics, or tracking algorithm. The four datasets contain trajectories of histone protein H2B, transcription factors CTCF and Sox2 and a protein consisting of three tandem repeats of nuclear localization signal (3 x NLS), all fused to a HaloTag. What makes these datasets interesting for DL-MSS analysis is that they range from being mostly stuck to being mostly free, in the order H2B – CTCF – Sox2 – 3 x NLS (see Fig. 4G,H in^42^). This spectrum of different types of behavior should become visible after DL-MSS analysis through a shift from the immobile state to the free state. The kernel density estimation plots for the four datasets (Fig. 4) clearly illustrate that DL-MSS indeed picks up the shift in *S*_*MSS*_-*D* space from immobile to free. The difference in *D* found for this publicly available dataset of H2B compared to the in-house H2B dataset from the previous showcase can be explained by the difference in frame rate for data acquisition (5 vs 30 ms interval, respectively). A higher frame rate means that interframe displacements can be smaller, while the detection error remains the same. This means that the detection error gets larger relative to the displacements between frames, leading to an overestimation of the associated diffusion constant.

## Discussion

DL-MSS is a general method to analyze single particle trajectories through single time step classification and clustering of segmented tracklets in *S*_*MSS*_-*D* space. This procedure yields specific mobility classes, which was exemplified by the showcases presented in this paper. As opposed to traditional analysis methods, DL-MSS defines mobility clusters based on the diffusion constant as well as the type of mobility. Additionally, this method makes use of state-of-the-art deep learning techniques for classification, which not only makes it possible to accurately segment trajectories into tracklets before calculating any biological parameters, but also allows classification of tracklets that would ordinarily be too short for mobility analysis. Moreover, DL-MSS is flexible, meaning that mobility classification is not restricted to either the number of classes the network was trained with, or the parameters (*D*, *S*_*MSS*_) that were assigned to the training data. Finally, this method is user-friendly; results can be obtained by running one single script, while still allowing the user to supervise all intermediate steps in classification and further analysis.

All these properties of DL-MSS can facilitate new insights into biological problems. In the example of BRCA2, it was already shown that this protein has multiple states of mobility^12^. However, instead of fitting a certain number of diffusion classes, DL-MSS yields new information about the types of motion in BRCA2 behavior (one fast diffusive class, one slow subdiffusive class and one immobile class). Furthermore, it was shown that the mobility classes of BRCA2 do not change with regard to their location in *S*_*MSS*_-*D* space when DNA damage is introduced into the cells. Rather, it is the relative density of the three clusters that changes. Additionally, the flexibility of DL-MSS was illustrated by the showcases of H2B, NLS and Spot-On datasets. These results clearly showed that this method is not restricted to the classification of mobility patterns that exhibit behavior similar to the simulated data the network was trained with, irrespective of the methods that were used to obtain the trajectory data.

Altogether, DL-MSS is a very versatile method that can be used for a wide range of applications. Moreover, DL-MSS is not only useful to analyze different molecules separately, but also to compare mobility patterns between different types of molecules. By analyzing mobility classes from datasets obtained through different experiments, the meaning and function of those classes can be elucidated. This is especially interesting when certain molecules are suspected to interact with each other, when there are different variants of the same molecule, or when multiple datasets are available of the same molecule but within different environments or with different treatments. Comparing different datasets to one another can easily be done using DL-MSS, as different datasets can be fully analyzed in parallel in only a few minutes up to a few hours, depending on the size of the dataset and computing power. All datasets mentioned in this paper were analyzed within 1 hour on a normal laptop (1.8–2.4 GHz Intel i7 CPU with 8 GB RAM) with the exception of the larger Spot-On H2B dataset, which took 2.5 hours (Supplementary Note 10). Of course, this method requires training, which takes 2–3 hours on the GPU used in this study (Nvidia GTX 980), or 6–7 hours on the CPU used. However, the training has to be done only once, after which the model can be saved and applied as many times as needed.

The DL-MSS software is not limited to producing the type of results shown in this paper. Depending on the application and the needs of the user, the software can be used to extract additional useful parameters for the dataset and there is a large variety of visualization options (Supplementary Note 11). For example, classification results can be used to determine the switching probabilities from one state to another, the dwell times per state, and the fraction of time points spent in each state. In terms of visualization, molecule trajectories can be plotted inside the cell (nucleus) with different colors per state in order to see if there are certain patterns. In the example of BRCA2, this type of figure could be useful to see if there are regions inside the cell nucleus where more BRCA2 proteins are immobile, possibly indicating the presence of DNA damage in these regions. Additionally, DL-MSS can be used to detect inconsistencies in tracking, which manifest themselves as clusters at unexpected locations (Supplementary Note 12).

Since there are no clearly defined rules for deep learning, there are many ways to adapt and elaborate the network that is part of the DL-MSS method proposed in this paper to increase accuracy on simulated training data, where the ground truth is available. Theoretically it should even be possible to train a network that outputs the diffusion constant *D* and moment scaling spectrum slope *S*_*MSS*_ at once. However, this would likely lead to an increase in computational demand. The relatively straightforward network presented here offers a nice balance between simplicity, performance and flexibility. Moreover, the uncoupled MSS analysis gives the opportunity to monitor and control the output of the network. Overall, DL-MSS provides a new, robust and very flexible tool for particle mobility analysis.

## Online Methods

### Simulation of trajectories for training

The lengths of the simulated trajectories were randomly sampled according to ${L}_{{\rm{track}}} \sim Exp(\lambda )=\lambda {e}^{-\lambda x}$ with rate parameter *λ*^50^. Every track was randomly assigned an initial type of mobility and labeled correspondingly. The switching probability was modeled using a Markov model^51^ with state transition probability matrix

2 $${\boldsymbol{\Pi }}=[\begin{array}{ccc}{p}_{00} & {p}_{01} & \cdots \\ {p}_{10} & {p}_{11} & \cdots \\ \vdots  & \vdots  & \ddots \end{array}],$$

where *p*_*ii*_ is the probability of remaining in state *i* and *p*_*ij*_ is the probability of switching from state *i* to state *j*. With such a problem setup, the number of steps *S*_*i*_ that a particle will remain in a certain state *i* can be sampled using a geometric distribution, where $\Pr ({S}_{i}=k)={{p}_{ii}}^{k-1}(1-{p}_{ii})$ gives the probability that the *k*^th^ step is followed by a switching event. To create training sets, it should be possible to generate any type of process, diffusive as well as anomalous. Pure diffusion (Brownian motion) can easily be simulated in 1D from the normal distribution ${\mathscr{N}}(\mu ,\,{\sigma }^{2})$ with *μ* = 0 and $\sigma =\sqrt{2D\tau }$ (diffusion constant *D* and time step *τ*, where *τ* can be chosen but does not have to match the real data). For 2D or 3D cases, the simulation of displacements is done independently for each coordinate. Anomalous diffusion was modeled using fractional Brownian motion (fBm)^52^, where the type of motion depends on the Hurst component *H*, which is equal to 0.5 for pure diffusion, lower than 0.5 for subdiffusion and higher than 0.5 for superdiffusion. fBm can be simulated^53^ using

3 $$\Delta x(t)=\frac{{n}^{-H}}{\Gamma (H+\frac{1}{2})}\,(\mathop{\sum }\limits_{i=1}^{n}{i}^{H-\frac{1}{2}}\,{\xi }_{(1+n(M+t)-i)}+\mathop{\sum }\limits_{i=1}^{n(M-1)}{(n+i)}^{H-\frac{1}{2}}-{i}^{H-\frac{1}{2}}\,{\xi }_{(1+n(M-1+t)-i)})$$

where $\Delta x(t)$ is the displacement in *x* for one time step, *n* is the number of intervals that every time step is divided into, *H* is the Hurst component, $\varGamma$ is the gamma function, *t* is the integer time, *M* is the range that can be covered in time *t* and $\xi$ are independent and identically distributed samples from a normal (Gaussian) distribution with zero mean and unit variance. $\Delta y(t)$ was simulated in the same way as $\Delta x(t)$ to create any type of anomalous diffusion as well as pure diffusion (Supplementary Note 13). The data was subsequently scaled to appear at specific *S*_*MSS*_ versus *D* using scaling factor *η*. For the model used in this paper, training was done with three-state simulated data with three corresponding labels: “$0$” for diffusion with $D\,=\,1.0\,{\rm{\mu }}{{\rm{m}}}^{2}/{\rm{s}}$, “$1$” for diffusion with $D\,=\,0.1\,{\rm{\mu }}{{\rm{m}}}^{2}/{\rm{s}}$ and “$2$” for the immobile state with Hurst coefficient $H\,=\,0.1$ and scaling factor $\eta \,=\,0.3$. The transition probability matrix is given by:

4 $${\boldsymbol{\Pi }}=[\begin{array}{ccc}0.8 & 0.1 & 0.1\\ 0.1 & 0.8 & 0.1\\ 0.1 & 0.1 & 0.8\end{array}].$$

### Deep learning using an LSTM recurrent neural network

As mobility state prediction requires sequential analysis as well as the ability to learn long-term dependencies, the model of choice for DL-MSS was a bidirectional Long Short Term Memory (LSTM) network^45,54^. Using a bidirectional network increased the performance of both ends of the window as well as in the middle compared to only forward and only reverse networks (Supplementary Note 14). For each time step, the distance travelled by the molecule was fed into the network, along with the x- and y- coordinates of the two points flanking this distance and some higher order average distances (Supplementary Note 15). The number of LSTM units corresponds to the number of time steps in a trajectory and every unit outputs a class label by passing the resulting hidden state (containing $200$ hidden units, Supplementary Note 16) through a fully connected layer before passing the hidden state on to the next unit. The model was implemented in Keras (with TensorFlow as backend) and optimized using categorical cross entropy as the loss function and Adam as the optimization method^55,56^. EarlyStopping, an algorithm that stops the training process when the validation error considerably exceeds the training error, was used as a generalization method^57^.

The simulated trajectories were split into time windows of $25$ frames (Supplementary Note 16) and used for training ($10,000$ windows), validation ($5,000$ windows), and testing ($5,000$ windows) with a batch size of $\,256$. Ten “splits” were performed to get a reliable estimate for the accuracy of prediction, meaning that new training, validation and test sets were picked ten times from the total pool of available time windows to repeat the training procedure. The trained network can be applied to trajectories of any size.

### MSS analysis

For moment scaling spectrum (MSS) analysis, only tracklets that have a length of ten or more frames and do not have a negative $D$ or ${S}_{MSS}$ due to unstable linear regression were selected to get a reliable result. For kernel density estimation (KDE) on the data points in *S*_*MSS*_-*D* space, a Gaussian kernel was used with a bandwidth (${\rm{bw}}$) corresponding to Scott’s rule (${\rm{bw}}=\,{n}^{-1/(d+4)}$^58^, where *n* is the number of data points and *d* is the number of dimensions).

### DL-MSS software

The DL-MSS method was implemented in the Python programming language with Keras and using the TensorFlow library as the deep-learning backend. The software and other scripts used in the presented experiments are publicly available at GitHub: https://github.com/ismal/DL-MSS.

### Single molecule tracking experiments for BRCA2, H2B-HaloTag and HaloTag-NLS

IB10 mouse embryonic stem cells (mESCs) were cultured on gelatinized plates (0.1% porcine gelatin (Sigma)) in 50% DMEM (High-Glucose, Ultraglutamine, Lonza), 40% BRL conditioned medium and 10% FCS supplemented with non-essential amino acids, 0.1 mM β-mercaptoethanol, pen/strep and 1,000 U/ml leukemia inhibitory factor.

BRCA2 in these mESCs was tagged with HaloTag at the C-terminus by modification of the endogenous locus of BRCA2 using CRISPR/Cas9. A detailed description of the exact methods can be found in^59^. In short, cells were electroporated with 15 µg of both the px459 Cas9/gRNA plasmid (gRNA: gctgttgagtcttagcctcc) as well as the donor plasmid consisting of homology arms and HaloTag-F2A-neo cassette^12^. After antibiotic selection, clones were picked and validated for homozygous integration of the cassette by PCR genotyping and western blotting. H2B-HaloTag and HaloTag-NLS were cloned into a PiggyBac vector^60^ containing a CAG promoter and PGK-puro selection cassette, and stable cell lines were generated by Lipofectamine 3000 transfection followed by puromycin selection.

For imaging, cells were seeded in µ-Slide 8 Well Glass Bottom (Ibidi) coated with 25 ug/ml Laminin (Roche) the day before the experiment. For ionizing radiation (IR) cells were treated with $5$ Gy of X-rays. Cells were labeled with fluorescent 5 nM JF549-HaloTag^61^ ligand (500 pM for HaloTag-H2B and HaloTag-NLS) for 15 minutes in Fluorobrite medium (ThermoFisher), complemented with 10%FCS, non-essential amino acids, 0.1 mM β-mercaptoethanol, pen/strep and 1,000 U/ml leukemia inhibitory factor. To remove free HaloTag ligand from the cells, twice Fluorobrite medium was exchanged with 15-minute interval. Experiments were done around 2 hours after irradiation. Imaging was performed using HiLo illumination on an Elyra PS1 system with 100 × 1.49NA α Plan Apochromat DIC (Zeiss) TIRF objective and Tokai Hit stage and objective heating (37 °C and 5% CO_2_). For excitation of JF549 a 100 mW 561 nm laser was used with a 570–650 nm bandpass filter. Signal was detected on an Andor iXon DU897 with 256 × 256 pixel region at 32 ms interval with an EMCCD gain of 300. In total 2000 frames were recorded per cell. JF549-HaloTag ligand was a kind gift from Luke Lavis.

A software tool (plug-in for ImageJ, which is publically available at http://smal.ws/wp/software/sosplugin/), to extract the protein trajectories from microscopy data for this application already existed in our group^62,63^. This algorithm finds the bright spots that represent single molecules in every time frame, fits a 2D Gaussian-like intensity profile to these spots and then constructs tracks by connecting spots from different time frames together through nearest-neighbor linking.

### Single molecule tracking experiments for Spot-On datasets (H2B, CTCF, Sox2 and 3xNLS)

The Spot-On publicly available datasets were acquired from HaloTag-Sox2 knock-in mESCs and HaloTag-3xNLS, H2B-HaloTag-SNAP and C32 HaloTag-CTCF knock-in human U2OS osteosarcoma cells (H2B, CTCF and 3 × NLS). Cells were labelled with PA-JF_646_ dye and imaged in phenol red-free medium at $37$ °C and $5 \%$ CO_2_ using TIRF microscopy. The datasets chosen for this paper were imaged at a frame rate of $201$ Hz (time step of $5$ ms and pixel size of $0.16$ µm) and are available at https://zenodo.org/record/834781#.XC-YHlVKjX6. Molecules in these datasets were tracked using a custom-written Matlab implementation of the MTT-algorithm^64^. Additional information on data acquisition and single molecule tracking for the Spot-On datasets (H2B, CTCF, Sox2 and 3xNLS) can be found in^42^.

## Supplementary information

Supplementary Information
