# Supplementary material for: Particle Mobility Analysis Using Deep Learning and the Moment Scaling Spectrum
Source: Sci Rep. 2019 Nov 20;9:17160. doi: 10.1038/s41598-019-53663-8 (PMC6868130; doi:10.1038/s41598-019-53663-8)
Supplement: Supplementary file 1 — Supplementary Information [file 41598_2019_53663_MOESM1_ESM.pdf]

Particle Mobility Analysis Using Deep Learning and the Moment Scaling Spectrum

Marloes Arts, Ihor Smal, Maarten W. Paul, Claire Wyman, Erik Meijering

Supplementary Note 1: Limitations of PDF/CDF fitting

One of the single-step approaches to characterize motion is fitting a PDF or CDF over the histogram of displacements. For diffusive processes, it is known that displacements per time step follow the Rayleigh distribution:

$$\mathrm{PDF}_{\mathrm{Rayleigh}} = \frac{d}{\sigma^2} e^{-\frac{d^2}{2\sigma^2}}$$

$$\mathrm{CDF}_{\mathrm{Rayleigh}} = 1 - e^{-\frac{d^2}{2\sigma^2}}$$

with displacements d per time step τ and $\sigma = \sqrt{2D\tau}$ for Brownian motion in one dimension (because displacements are considered instead of coordinates). These distributions can be fit over the displacement histogram of the data to get an estimate for D. For example, this can be done for simulated two-state diffusive data (with $D_{\mathrm{slow}} = 0.1\mu m^2/s$ and $D_{\mathrm{fast}} = 1.0\mu m^2/s$) using the `lmfit` package in Python (**Figure S1a,b**). PDF and CDF fitting lead to quite accurate estimations of D for this simulated data that is purely diffusive. Even though tools based on PDF or CDF fits are a robust way to separate populations of diffusing molecules, they will not give accurate results for other types of motion or for an unknown number of subpopulations. As an example, fitting two subpopulations to three-state simulated data ($D = 1.0\mathrm{\mu}m^2/s$ for the fast state, $D = 0.1\mathrm{\mu}m^2/s$ for the slow state, and $H = 0.1, \eta = 0.3$ for the immobile state) does not give accurate results (**Figure S1c,d**). Moreover, PDF and CDF fitting does not provide any information about mobility switching of particles.

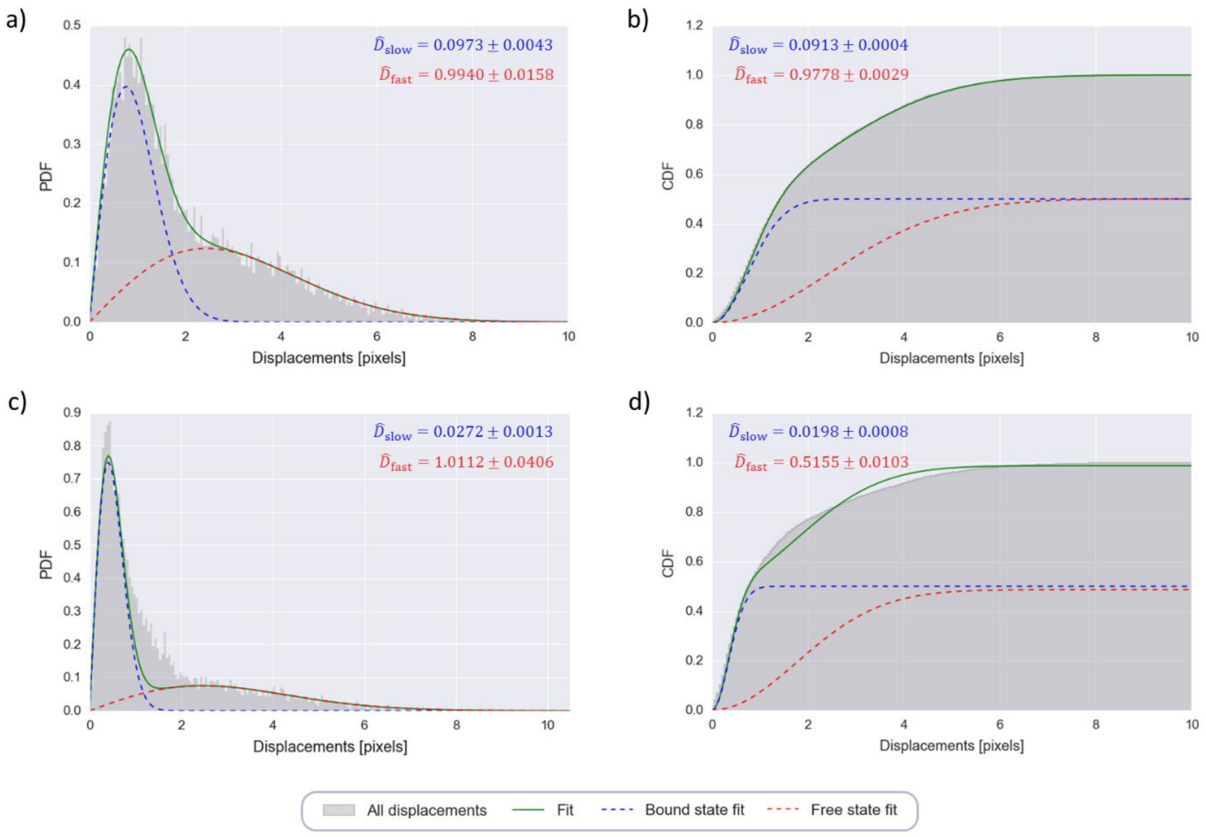

Figure S1. Example of PDF (left) and CDF (right) fits on displacement histograms for a, b: simulated two-state diffusion data with $D_{slow} = 0.1\mu m^2/s$ and $D_{fast} = 1.0\mu m^2/s$ and c, d: simulated three-state data with $D = 1.0\mathrm{\mu}m^2/s$ for the fast state, $D = 0.1\mathrm{\mu}m^2/s$ for the slow state, and $H = 0.1$, $\eta = 0.3$ for the immobile state.

Supplementary Note 2: Limitations of MSD-based analysis

Most methods traditionally used in biological applications of single particle tracking are based on mean square displacements (MSD):

$$\mathrm{MSD}(n\tau) = \frac{1}{N+1}\sum_{i=0}^{N}([x_{i+n} - x_i]^2 + [y_{i+n} - y_i]^2)$$

where $n = 0, 1, 2, \ldots$, and τ is the data acquisition time interval, N is the number of available windows for the given n, and x and y are coordinates. For pure diffusion, exhibiting Brownian motion (BM) in two dimensions (2D), it holds that:

$$\mathrm{MSD}_{\mathrm{BM(2D)}} = 4D\tau$$

with diffusion constant D. Because of this known relationship between MSD and τ, the plot of MSD versus τ will be a straight line and the diffusion constant can be determined from the slope of the line, which is equal to $4D$. Therefore, by fitting a straight line through the MSD measurements of every trajectory, an approximation of the diffusion constant of that trajectory can be calculated. The values of the logarithm of the diffusion constants of each particle trajectory can subsequently be plotted in a histogram to see if there are distinguishable populations (the logarithmic scale is used to magnify the distinction between peaks). This can for instance be done for a BRCA2 dataset (**Figure S2**), resulting in two observable peaks in the MSD histogram. A large drawback of this method is that trajectories that switch between mobility classes are being treated as if they exhibit only one type of motion, leading to an inaccurate estimation of D for each mobility class. Additionally, this method does not take into account motion types that are not diffusive.

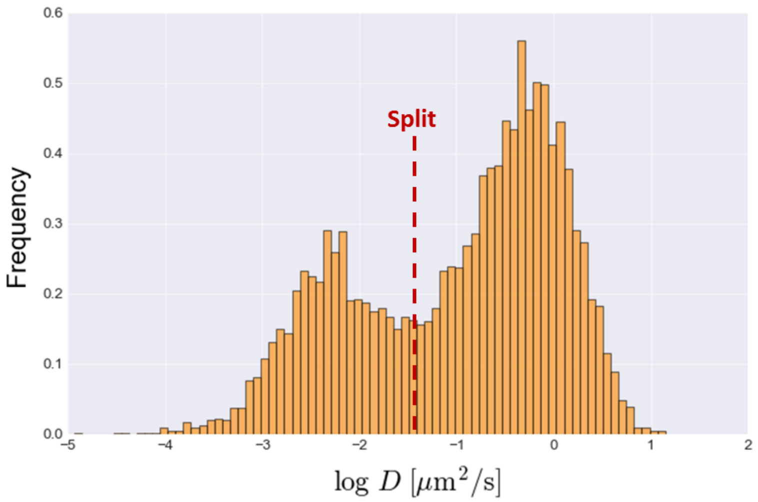

Figure S2. An example of a histogram showing the frequency of diffusion constants that were estimated per BRCA2 trajectory using MSD on a logarithmic scale. There seem to be two populations: one with a higher diffusion constant and one with a lower diffusion constant. The red dotted line indicates the splitting point.

Supplementary Note 3: Long short term memory network

Long short term memory networks (LSTMs) are sophisticated recurrent neural networks that can selectively "remember" some past events and "forget" others over (iteration) time t (**Figure S3**). Besides a hidden output state h_t, LSTMs also maintain a cell memory state C_t that allows the network to keep track of what is important to remember and what to forget. This idea is realized by gates that can selectively let information through. These gates are the forget gate f_t, the input gate i_t and the output gate o_t. By using these gates, LSTMs manage to get only relevant information to pass through, enabling them to learn long-term dependencies.

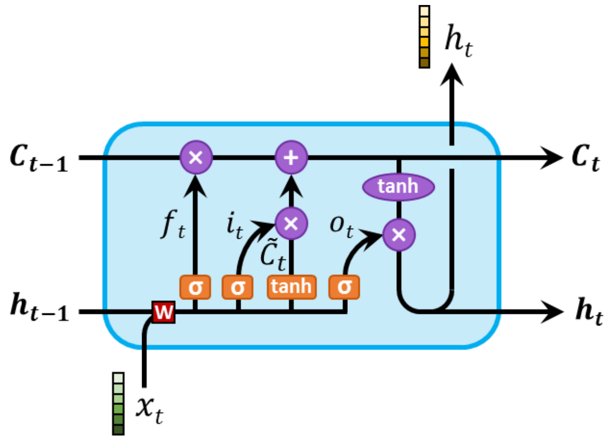

Figure S3. Schematic representation of an LSTM network unit. Neural network layers are shown in orange, point-wise operations are shown in purple. Red blocks with W indicate weight matrices that are trained. h: hidden state, x: input, $tanh$: hyperbolic tangent, σ: sigmoid, C: cell memory state, f: forget gate, i: input gate, $\tilde{C}$: potential cell state update value and o: output gate.

Supplementary Note 4: DL-MSS does not detect redundant clusters

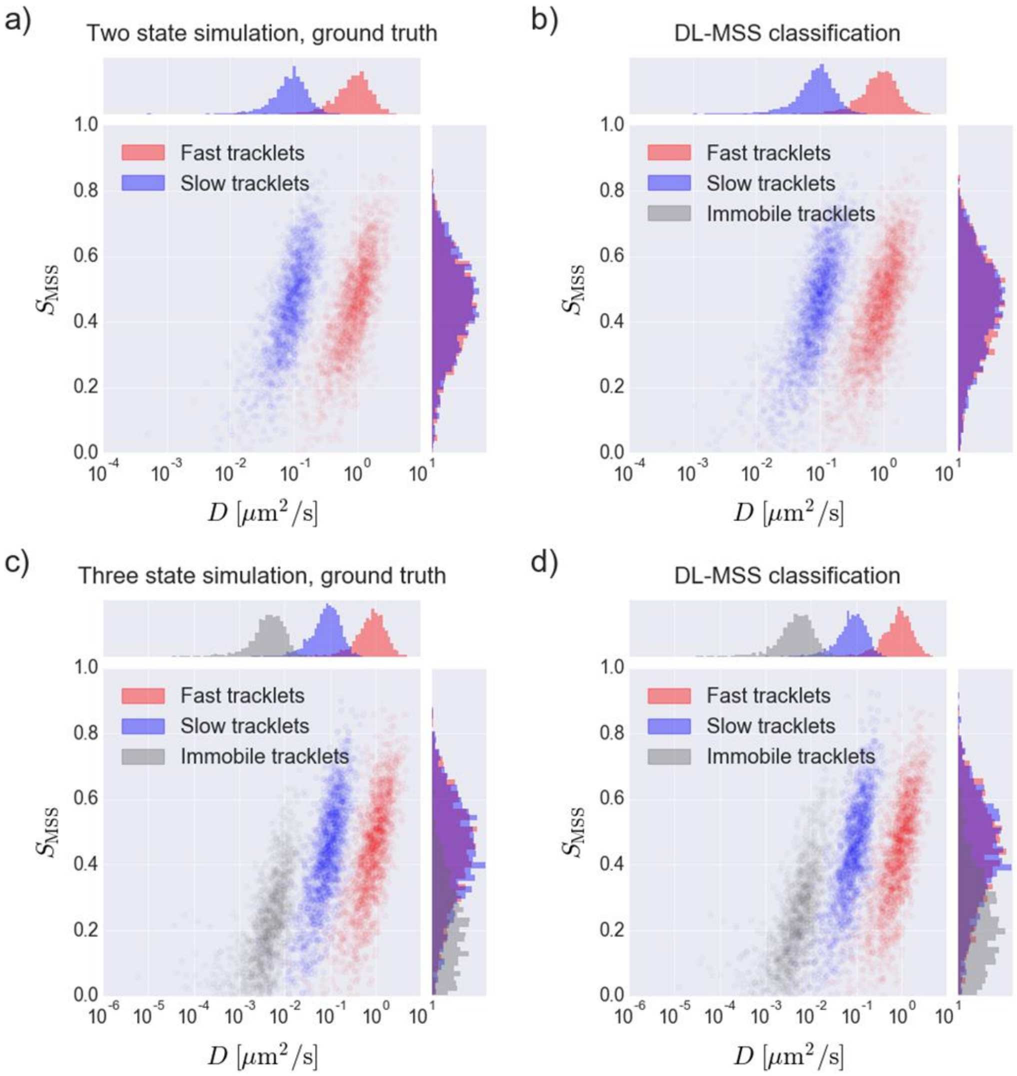

Figure S4. Even though the proposed version of DL-MSS is trained with three states of mobility, this does not mean that the method will always detect three clusters irrespective of the dataset being analyzed. When a certain mobility type is not present, this cluster will not appear. a, b: The classification result of DL-MSS on two-state simulated data (**Online Methods**) corresponds with the ground truth, no immobile cluster is detected. c, d: DL-MSS accurately finds three mobility clusters for three-state simulated data (**Online Methods**).

Supplementary Note 5: DL-MSS is able to detect extra clusters

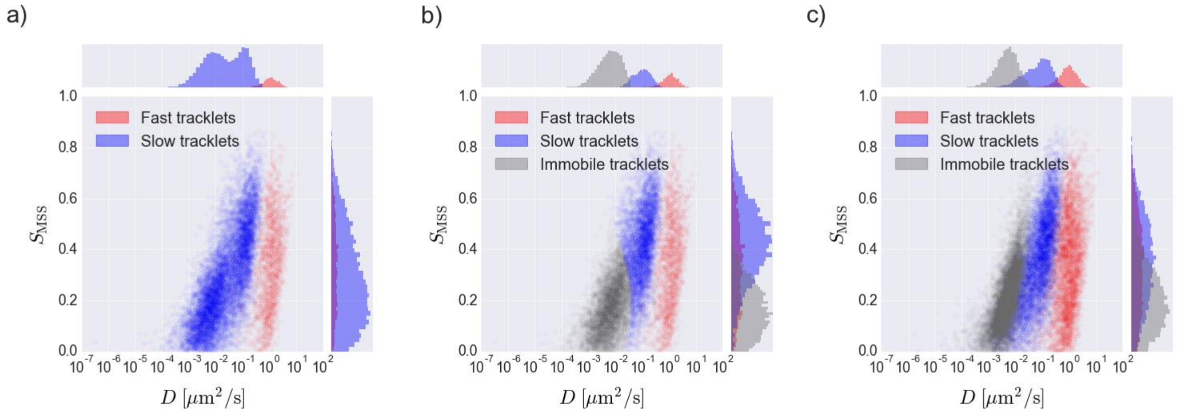

Figure S5. DL-MSS is able to pick up extra clusters. a: A network was trained with two-state simulated data and applied to three-state data (**Online Methods**). It is clear that there are two clusters present in the slow class. b: The slow cluster can be split into separate clusters in a simple way using Gaussian fitting. However, to get accurate results for tracklets that switch between immobile and slow (or even between immobile and fast), a network that was trained with three states needs to be applied. c: A network that was trained with three-state data yields the same type of pattern, but the clusters are more well-defined.

Supplementary Note 6: Examples of trajectory classification

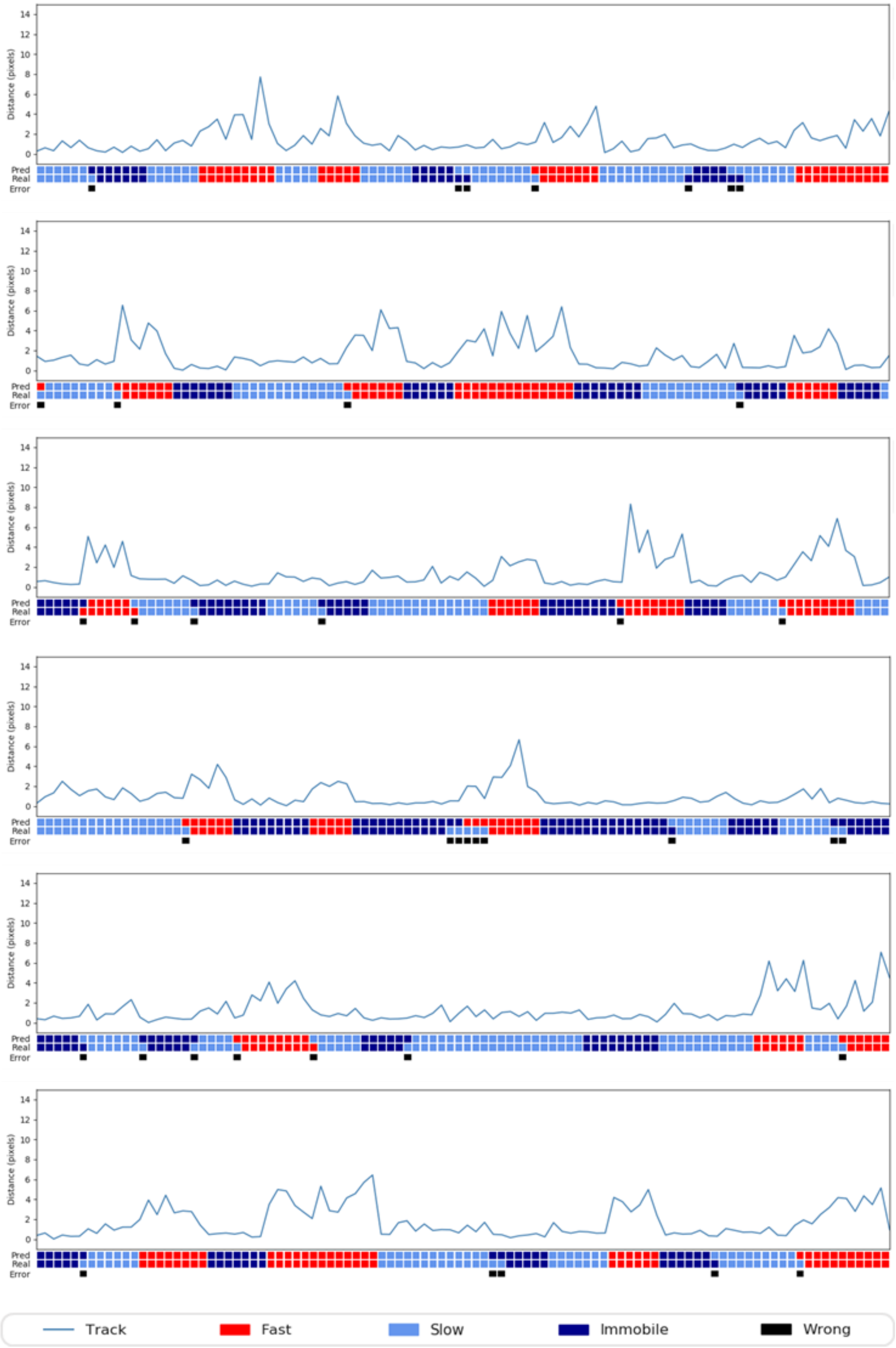

Figure S6. Examples of trajectory classification by the LSTM network on simulated data (with ground truth available). Travelled distances of the particle are shown per time point for a hundred frames, along with the predicted class and the real class. Note: classification mistakes are mainly made at the ends of tracklets, meaning that tracklets are seldom broken up by wrongly classified states.

Supplementary Note 7: Regions in moment scaling spectrum analysis

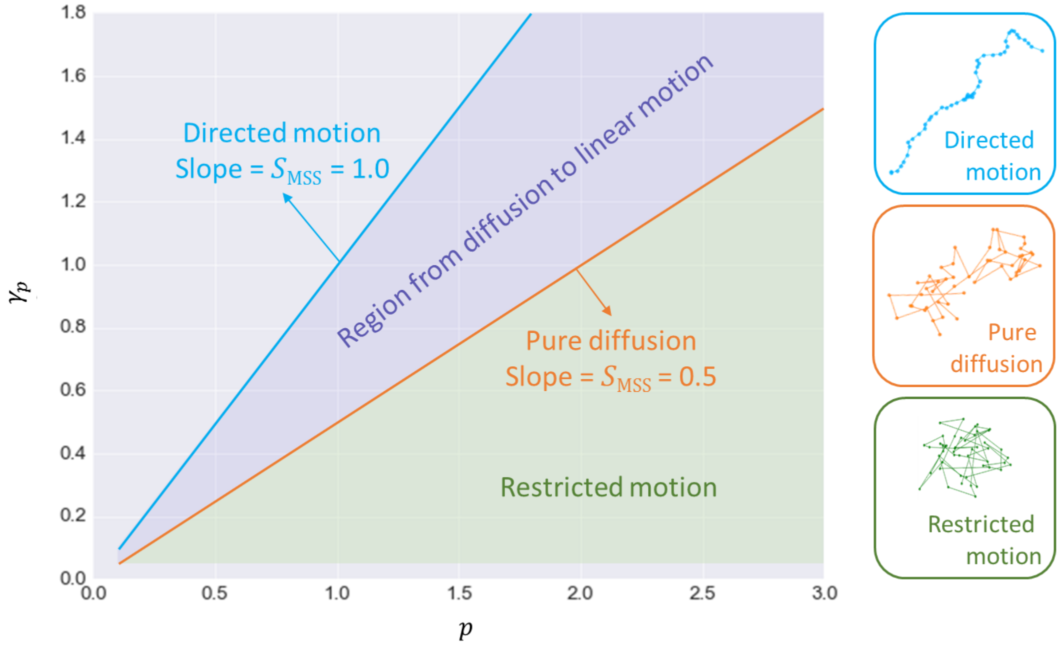

Figure S7. Regions in the moment scaling spectrum. An $S_{MSS} = 0.5$ represents pure diffusion, $0 < S_{MSS} < 0.5$ represents restricted motion and $0.5 < S_{MSS} < 1.0$ represents more directed motion. Some visual examples of different types of trajectories are shown on the right of the spectrum.

Supplementary Note 8: Use of $\boldsymbol{D}$ as an invariable measure (irrespective of moment order $\boldsymbol{p}$)

In the main text, clustering of tracklets is done in S_{MSS}-D space, where S_{MSS} indicates the type of motion and D is used to distinguish between "faster" and "slower" motion. Intuitively, it might seem strange to use this measure, which characterizes diffusion specifically and is traditionally calculated using the second order moment only. Here it is shown that the diffusion constant is invariant over the moment order and can be used as a measure for subdiffusive (immobile) tracklets as well.

First of all, the diffusion constant can also be calculated from other moments. The moments μ_p can be calculated as

$$\mu_p = \int_{-\infty}^{\infty} d^p \ \mathrm{PDF}(d) \ \mathrm{d}d,$$

with d displacement, p moment order, and $\mathrm{PDF}(d) = \frac{d}{\sigma^2} e^{-\frac{d^2}{2\sigma^2}}$ because the displacements are Rayleigh distributed, where $\sigma^2 = 2D\tau$ is the variance (D diffusion constant, τ time step). This integral can be solved analytically to obtain

$$\mu_p = \sigma^p 2^{p/2} \ \Gamma\left(1 + \frac{p}{2}\right).$$

Here, $\Gamma(\mathrm{n}) = (\mathrm{n} - 1)!$ is the gamma function. Finally, this result can be rearranged to obtain a formula for D as a function of p:

$$D(p) = \left(\frac{\mu_p}{2^{p/2} \ \Gamma\left(1 + \frac{p}{2}\right)} \right)^{2/p} \Bigg/ 2\tau$$

When D is plotted against p for simulated three-state data, it becomes clear that the D is practically constant over p (**Figure S8**). Moreover, the diffusion constant is also constant for the immobile class, demonstrating that in combination with S_{MSS}, D can indeed be used as a measure to distinguish between fast diffusive, slow diffusive and immobile tracklets.

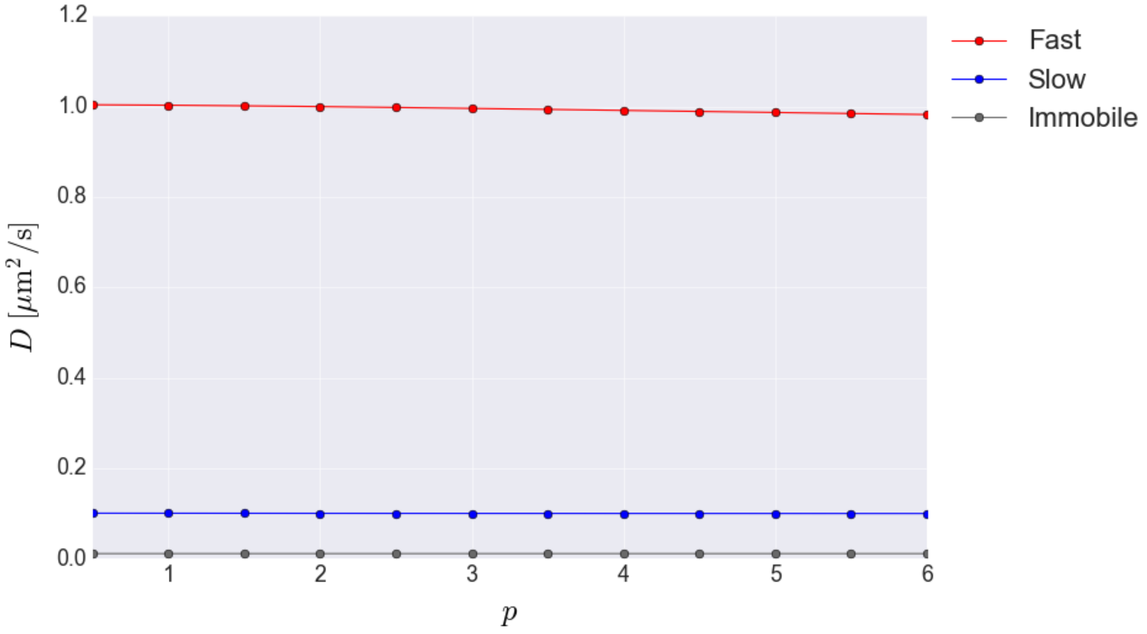

Figure S8. Diffusion constant D versus moment order p for different states in simulated data. D is practically constant over p.

Supplementary Note 9: Effect of tracklet size on clustering in $\boldsymbol{S_{MSS}}$-$\boldsymbol{D}$ space

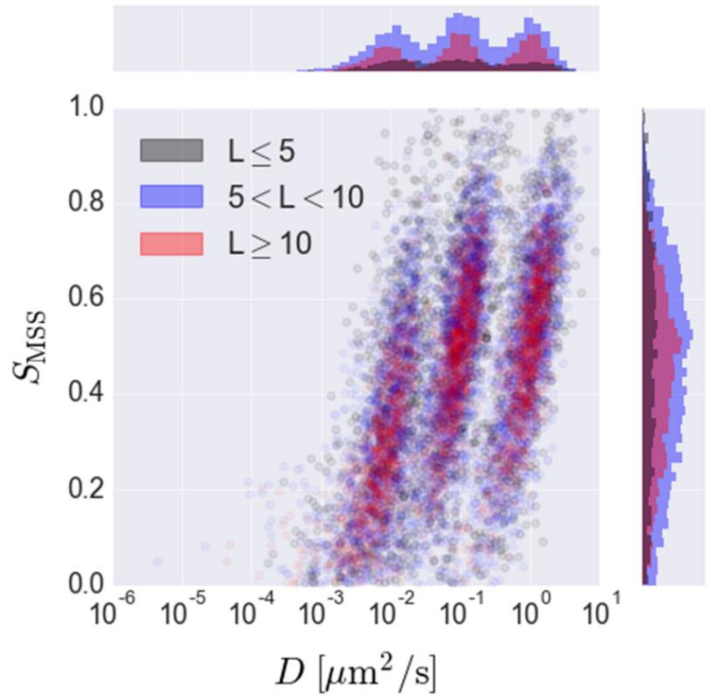

Figure S9. Effect of tracklet size (L) on the location in S_{MSS}-D space (in three-state simulated data). Smaller tracklets deviate more from the center of their corresponding cluster than larger tracklets. For easier and more accurate determination of mobility parameters, only tracklets of $L \geq 10$ are used for clustering.

Supplementary Note 10: Runtimes of the DL-MSS software

Table S1. Runtimes for different parts of the DL-MSS method on all datasets used in this paper. All times are given in the format hours : minutes : seconds. Run on 1.8-2.4 GHz Intel i7 CPU with 8 GB RAM.

Dataset	Initialization & importing model	Data loading	Classification	Total
BRCA2 -IR	00:02:00	00:01:42	00:00:36	00:04:18
BRCA2 +IR		00:02:28	00:00:49	00:05:17
H2B		00:01:21	00:00:21	00:03:42
NLS		00:07:35	00:00:37	00:10:12
H2B (Spot-On)		02:28:05	00:02:18	02:32:23
CTCF (Spot-On)		00:42:21	00:01:05	00:45:26
Sox2 (Spot-On)		00:29:09	00:00:57	00:32:06
3 x NLS (Spot-On)		00:28:14	00:00:34	00:30:48
**Average**		**00:32:37**	**00:00:55**	**00:35:32**

Supplementary Note 11: Examples of additional property analysis and visualization

The output of DL-MSS analysis is not limited to the type of results shown in the main text of this paper. This section shows some examples of additional data analysis and visualizations.

Firstly, the results of classification by the deep learning network can be used to calculate transition probabilities between the different classes of mobility (**Figure S10**).

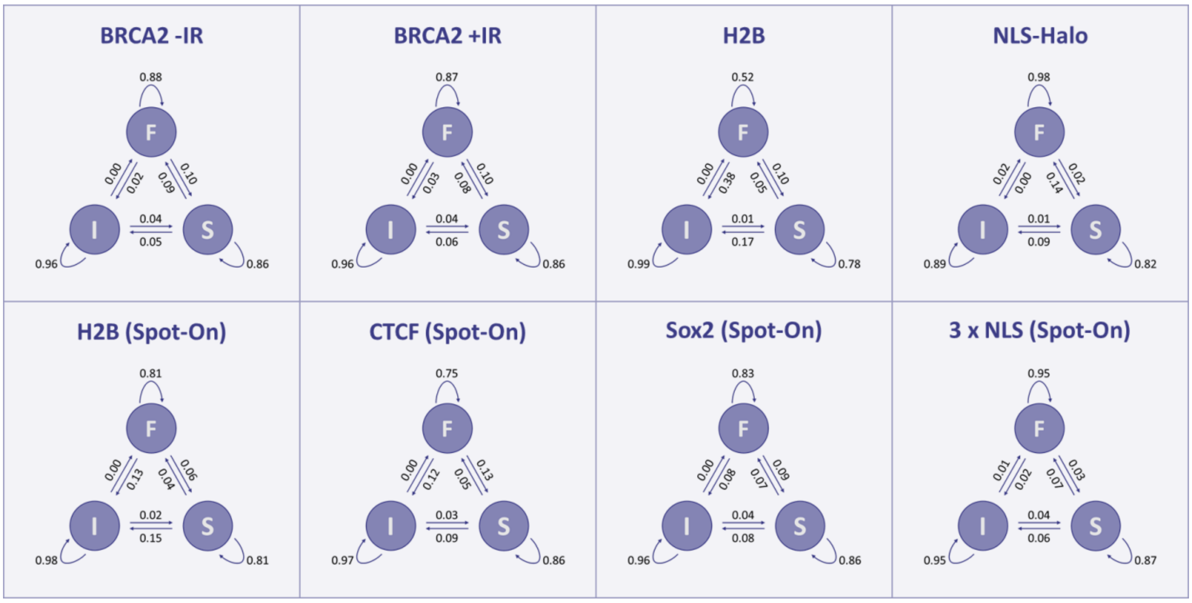

Figure S10. Diagrams of transition probabilities for all eight datasets mentioned in this paper. F = fast, S = slow, and I = immobile.

Secondly, depending on the research question, it may be interesting to know the mean dwell time of the particle in a certain state (**Table S2**). These dwell times can be derived only from those segments of the trajectories between any two successive points of switching between different motion types (the inner tracklets). This is because it is not known how long a particle was in the given motion state before entering or after leaving the field of view. Therefore, trajectories with less than two switch points, and the trajectory segments up to the first and after the last switch point (the outer tracklets), must be ignored. Even though this implies not all data can be used, the mean dwell times computed from the inner tracklets still provide useful information to compare different datasets. One thing that stands out when comparing the dwell times of the datasets used in this paper is that the Spot-On datasets have much shorter dwell times, probably due to the fact that the acquisition speed of the datasets (time step of 5 ms) is much faster than our in-house datasets (time step of 30 ms). For higher acquisition speeds, the probability to detect switches in type of mobility is larger, as switches might be “missed” when a lower acquisition speed is used.

Thirdly, it is possible to calculate the fraction of timepoints spent in each state (**Table S2**). This gives yet another measure that makes it easier to compare different datasets.

Table S2. Mean dwell times in each state and fraction of timepoints spent in each state for all eight datasets mentioned in this paper.

Dataset	Mean dwell times [ms]			Fraction of timepoints		
	**Fast**	**Slow**	**Immobile**	**Fast**	**Slow**	**Immobile**
**BRCA2 -IR**	141.8	165.4	359.0	0.26	0.30	0.44
**BRCA2 +IR**	137.4	165.0	376.4	0.21	0.26	0.53
**H2B**	56.8	89.9	284.6	0.01	0.05	0.94
**NLS-Halo**	326.0	155.4	255.9	0.81	0.11	0.08
**H2B (Spot-On)**	13.9	12.7	17.4	0.06	0.09	0.85
**CTCF (Spot-On)**	9.7	14.3	15.4	0.09	0.18	0.73
**Sox2 (Spot-On)**	9.5	14.3	15.0	0.20	0.21	0.59
**3 x NLS (Spot-On)**	9.5	15.2	14.6	0.57	0.16	0.27

Finally, visualizations in single cells could be useful, especially for biological applications. For instance, in the example of BRCA2 it would be useful to see if there are certain regions in the cell where BRCA2 is predominantly immobile, as this could indicate regions where there is more DNA damage to repair. These type of patterns can be uncovered by studying the distribution of different types of mobility inside the cell (**Figure S11**). For instance, tracklets can be colored by class (**Figure S11b**) or the density of displacements of different mobility types can be represented as a heat map (**Figure S11c,d,e**).

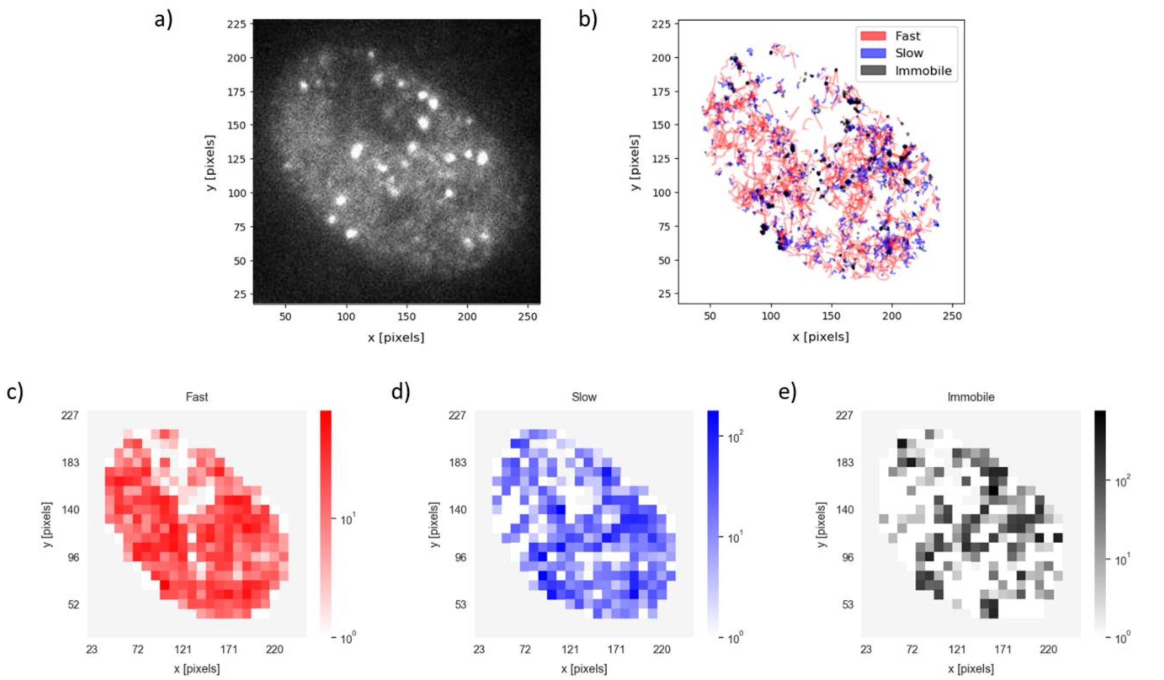

Figure S11. Single cell visualizations of DL-MSS results for a cell where BRCA2 was tracked. a: Fluorescence microscopy image of a cell, where every bright spot represents a fluorescently tagged BRCA2 protein (note: this is a snapshot, the location of the spots changes over time). b: Visualization of fast, slow and immobile tracklets inside the cell for the entire duration of imaging. c, d, e: Heat maps of displacements in fast/slow/immobile states, where a higher color intensity indicates more displacements in that state in a certain area. Pixelsize is 0.1 µm.

Supplementary Note 12: Uncovering tracking deficiencies using DL-MSS

DL-MSS can reveal inconsistencies in tracking by showing clusters with a deviating type of mobility. The first case that made this ability clear was a dataset of H2B trajectories that were tracked using settings optimized for BRCA2 tracking. For H2B trajectories, it is expected that nearly all trajectories exclusively exhibit the immobile class. When a scatterplot in S_{MSS}-D space was made for whole trajectories instead of tracklets (**Figure S12a**), there appeared an extra cluster that was faster than the immobile cluster (higher D), and even more confined (lower S_{MSS}). The scatterplot for the segmented tracklets (**Figure S12b**) showed that even though most tracklets were classified as immobile or slow, there were some unexpected fast tracklets as well. This was a sign that the trajectories in the strange additional clusters mainly consisted of immobile tracklets with only a few steps of faster, confined motion.

Revision of the H2B trajectories obtained from microscopy data indicated that some spots were wrongly linked from one time frame to another creating large displacements that should not be there, a problem that could be solved by adjusting the tracking parameters. After this adjustment, the strange cluster nearly disappeared (**Figure S12c**). The same effect can also be simulated by introducing a bigger displacement every few time frames ("big skips"), for example in pure diffusion (**Figure S12d**). This gives the same type of extra cluster for whole trajectories as the H2B molecules before adjustment of tracking parameters. Overall, this result indicated that DL-MSS analysis can help identifying defects in tracking.

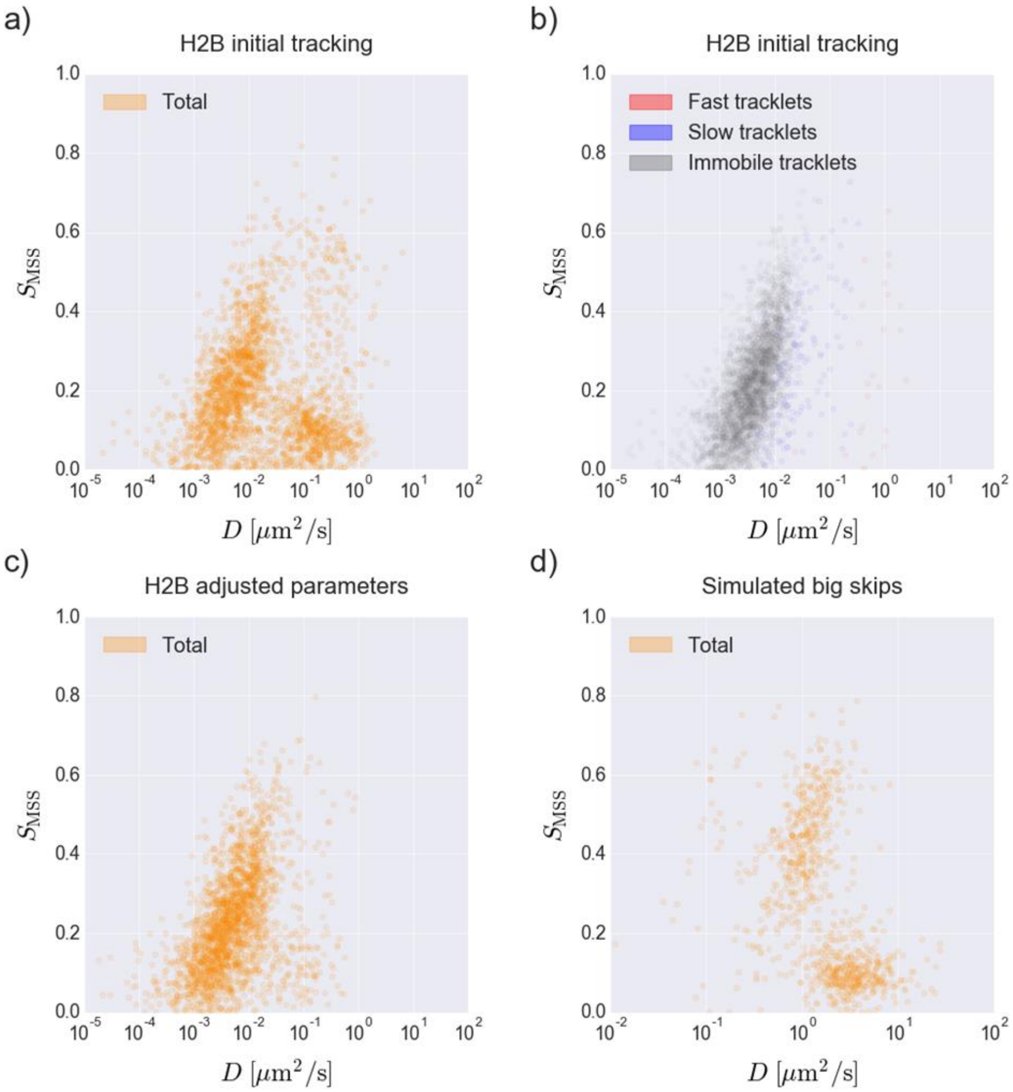

Figure S12. DL-MSS can be used to detect mistakes in tracking settings. a, b: Initial tracking with parameters optimized for BRCA2 resulted in an extra cluster of immobile tracklets with a few large displacements. a: Results for whole trajectories. b: Results for segmented tracklets. c: Adjustments of parameters resulted in a reduction of the extra cluster. d: The extra cluster can also be simulated by inserting a "big skip" every few time frames, in this case for diffusive motion.

Supplementary Note 13: Simulation examples

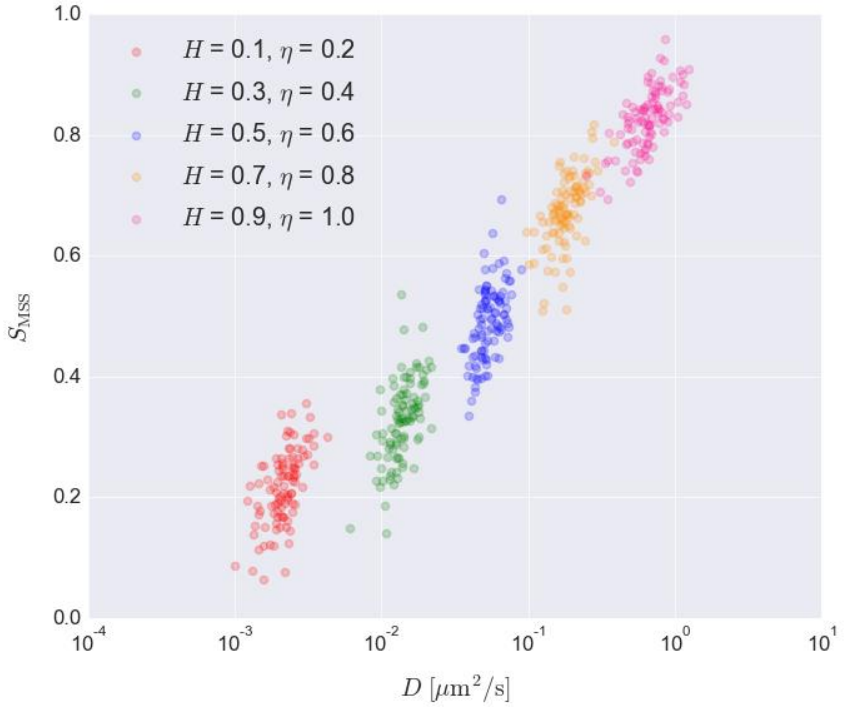

Figure S13. Examples of how data can be simulated with any desired S_{MSS} and D using Hurst component H and scaling factor η.

Supplementary Note 14: Bidirectional versus unidirectional networks

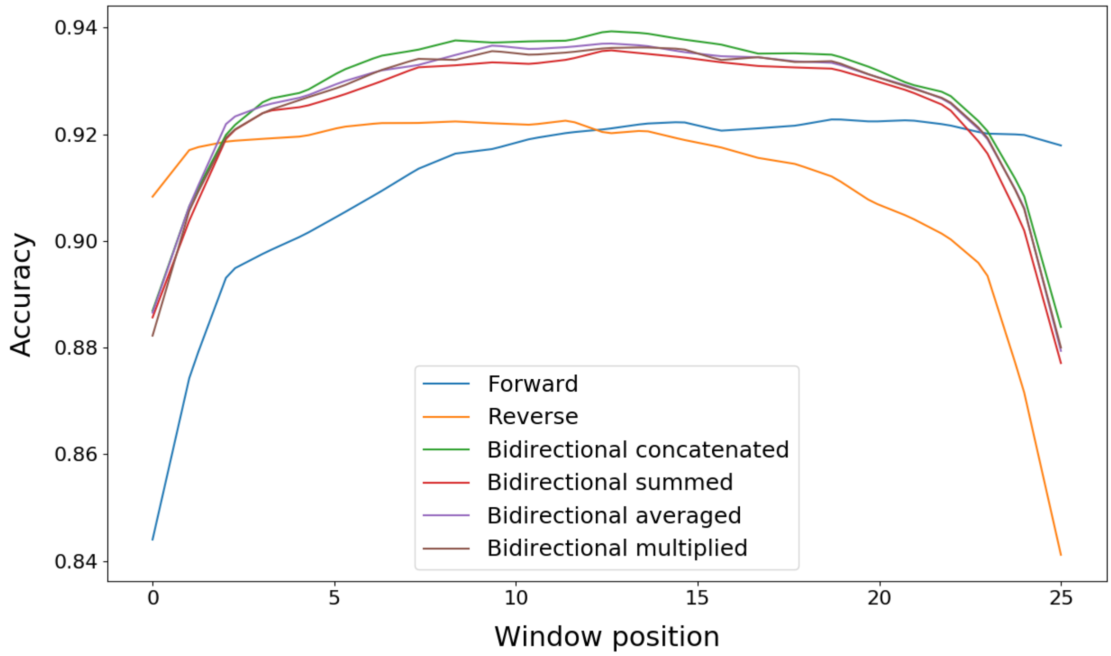

Figure S14. Using a bidirectional network increases the performance of both ends of the window as well as in the middle compared to only forward and only reverse networks. Hidden states from the forward and the reverse pass can be combined in several ways: by concatenation, summing, averaging or multiplication. These different bidirectional methods all perform similarly and are subject to statistical variance. For the method proposed in the main text, concatenation was chosen for the combination of hidden states as it is the most "unbiased" and intuitive option, where the network can learn how to combine the hidden states in the best possible way.

Supplementary Note 15: Data representation for input network

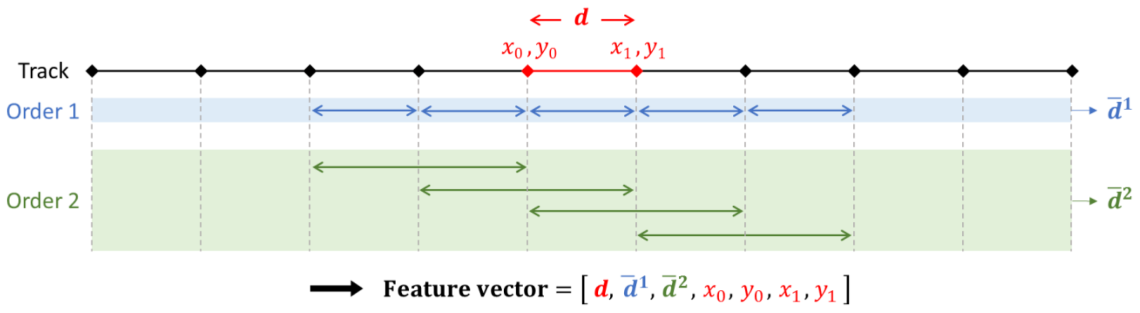

Figure S15. For every displacement fed into the network, a feature vector is provided containing the displacement d (calculated as $d_t = \sqrt{(x_t - x_{t-1})^2 + (y_t - y_{t-1})^2}$), the coordinates flanking the distance and some additional average displacements surrounding d, indicated as $\bar{d}^1$ and $\bar{d}^2$.

Supplementary Note 16: Choice of number of hidden units and window size

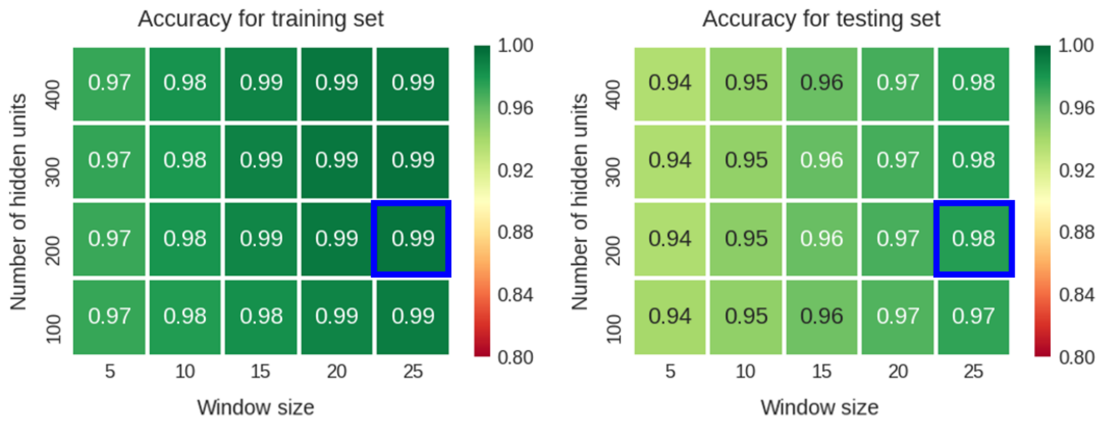

Figure S16. Examples of combinations of the number of hidden units and the window size that can be used, with corresponding accuracies on training and test sets. Color indicates the accuracy (see colorbar). For experiments, 200 hidden units were used in combination with a window size of 25 (indicated with blue squares). This combination was chosen because it gave very high accuracy without overtraining and does not require extensive computational power.
